# Negative pressure wound therapy promotes wound healing of diabetic foot ulcers by up-regulating PRDX2 in wound margin tissue

**DOI:** 10.1038/s41598-023-42634-9

**Published:** 2023-09-27

**Authors:** Ying Tang, Lei Liu, Ruyan Jie, Yizhong Tang, Xiaotong Zhao, Murong Xu, Mingwei Chen

**Affiliations:** 1 Department of Endocrinology, The First Affiliated Hospital of Anhui Medical University, No.218 Jixi Road, Shushan District, Hefei City, Anhui Province People’s Republic of China; 2 Department of Burns, The First Affiliated Hospital of Anhui Medical University, Hefei City, Anhui Province People’s Republic of China

**Keywords:** Cell biology, Immunology, Endocrinology

## Abstract

To understand the changes in the peroxiredoxin-2 (PRDX2) expression level in the wound margin tissue (T-PRDX2) of patients with diabetic foot ulcer (DFU) before and after negative pressure wound therapy (NPWT). Additionally, the study aimed to explore the association between PRDX2 expression and the treatment outcome of DFUs to provide a new theoretical basis for revealing the mechanism of NPWT promoting the healing of DFUs. Fifty-six type 2 diabetes patients with foot ulcers undergoing NPWT (the DFU group) and 28 patients with chronic lower limb skin ulcers with normal glucose tolerance undergoing NPWT (the skin ulcer control [SUC] group) were included in the study. T-PRDX2 was detected using Western blotting, and the superoxide dismutase (SOD) activity and the malondialdehyde (MDA) and glutathione (GSH) levels were detected using a biochemical method. In addition, in vitro experiments were conducted to determine the effect of PRDX2 expression on normal human dermal fibroblast (NHDF) proliferation, migration, and apoptosis. Before NPWT, the DFU group exhibited a significantly lower T-PRDX2 expression level compared with the SUC group. After one week of NPWT, the T-PRDX2 expression level, SOD activity, and GSH content in the wound margin tissues of the DFU and SUC groups significantly increased compared with the before NPWT levels. Conversely, the inflammatory indicators (white blood cell, neutrophil percentage, C-reactive protein, and procalcitonin) and MDA content were significantly lower than the before NPWT levels. The expression changes of T-PRDX2 before and after NPWT in the DFU and SUC groups were positively correlated with the 4-week wound healing rate. In vitro experiments demonstrated that PRDX2 could alleviate the oxidative stress in NHDFs, thereby promoting their proliferation and migration, while reducing cell apoptosis. NPWT promotes DFU healing by increasing T-PRDX2, and changes in the T-PRDX2 might be associated with the therapeutic effect of NPWT.

## Introduction

Diabetic foot presents a significant risk for disability in individuals with diabetes, with an associated risk of 25%^1^, which can cause ulcers, infections, and gangrene. Among the various manifestations of diabetic foot, diabetic foot ulcer (DFU) is particularly prevalent, with a global incidence rate of 6.3%^2^. DFU represents a complex and critical clinical challenge frequently observed in individuals with type 2 diabetes mellitus (T2DM), often leading to hospitalisation^3^. Additionally, due to its considerable treatment expenses, DFU imposes a substantial economic burden on patients' families and society^4^. Consequently, finding efficient treatment strategies for DFU and improving patient survival rates have become pressing issues demanding immediate attention.

The impaired healing of diabetic skin wounds is primarily attributed to prolonged glucose and lipid metabolic disorders, which aggravates local histiocyte oxidative stress and inflammatory reactions. This results in delayed resolution of wound inflammation, hindered microangiogenesis, and decreased extracellular matrix synthesis and deposition^5^. Notably, a study revealed that increased oxidative stress might adversely affect peripheral nerve blood supply, structure, and metabolism, resulting in the extensive deterioration of the peripheral nervous system^6^. Additionally, excessive production of reactive oxygen species (ROS) is involved in the onset and progression of various complications such as diabetes retinopathy and diabetes myocardial microvascular disease^7^. Peripheral neuropathy and microvascular disease are crucial mechanisms underlying the onset and progression of DFU^8^. Therefore, the key to delaying the progression of DFU and promoting DFU wound healing lies in mitigating oxidative stress.

Peroxiredoxin-2 (PRDX2) is a member of the peroxiredoxin family and is widely expressed in mammalian cytoplasm and various tissues^9^. Its crucial function lies in eliminating hydrogen peroxide and ROS^10^, thereby participating in the regulation of ROS-induced cell apoptosis, cell cycle blockade, and cell proliferation and differentiation^11^. Notably, an animal model experiment confirmed the protective role of PRDX2 as a ROS scavenger in atherosclerosis progression by inhibiting phenotypic changes in vascular smooth muscle cells through the mitogen-activated protein kinase signalling pathway^12^. Chen et al. found that PRDX2 mediates the protective effects of isorhapontigenin on heart microvessels in individuals with diabetes by inhibiting oxidative stress, iron overload, and lipid peroxidation, thus inhibiting mitochondrial-related iron removal^13^. Experimental studies have revealed that PRDX2 exhibits higher expression in benign vascular tumours compared with malignant vascular tumours such as Kaposi’s sarcoma and angiosarcoma. Its presence offers effective protection by mitigating oxidative damage caused by ROS in vascular endothelial cells, making it a novel marker for vascular tumours^14^. Furthermore, studies have demonstrated that the absence of PRDX2 could lead to abnormal Wnt/β-catenin signalling pathway activation, resulting in premature ageing of dermal mesenchymal stem cells, subsequently causing premature skin ageing and impaired wound healing in PRDX2 knockout mice^15^. These findings suggest that PRDX2 might promote wound healing through its antioxidative stress properties.

The process of skin wound healing is relatively complex, encompassing distinct stages including coagulation, inflammation, proliferation, and remodelling^16^. Successful wound healing necessitates the coordinated involvement of various cells and growth factors such as vascular endothelial cells, skin fibroblasts, and keratinocytes^17^. Recent studies have revealed that higher glucose concentrations (50 and 75 mM) exert a significant inhibitory effect on fibroblast proliferation, and cell proliferation and wound closure was the slowest in cells grown at 75 mM glucose^18^. Animal experiments have demonstrated that hyperglycaemia reduces the integrin receptor expression on the surface of rat dermal fibroblasts, impairing their migration ability, thereby affecting wound healing^19^. Additional studies have demonstrated that under high glucose (HG) conditions, the proliferation of skin fibroblasts is significantly reduced, while cell apoptosis is significantly increased^20, 21^. These findings indicate that HG levels could affect fibroblast function, thereby affecting wound healing.

In recent years, negative pressure wound therapy (NPWT) has emerged as a non-invasive wound healing technology widely employed for treating DFUs. NPWT aids in reducing inflammatory exudate and promotes granulation tissue growth by applying subatmospheric pressure, thereby optimising wound healing^22^. However, the mechanism by which NPWT promotes DFU wound healing is yet to be elucidated. Previous studies have used label-free quantitative mass spectrometry to analyse changes in protein expression within the wound margin tissue of three patients with DFUs before and after NPWT. These investigations revealed a significant up-regulation of the PRDX2 protein following NPWT, with preliminary validation obtained from the wound margin tissue of eight patients with DFUs^23^. These findings suggest that PRDX2 might be involved in the mechanism through which NPWT promotes DFU wound healing. Furthermore, previous studies have reported the potential of NPWT in promoting the proliferation and migration of wound fibroblasts^24^, albeit the precise underlying mechanism remains unclear. Therefore, this study aimed to propose and confirm that NPWT promotes fibroblast proliferation and migration by up-regulating PRDX2 expression, thereby affecting DFU healing.

## Material and methods

### Clinical study participants

**Figure 1:**
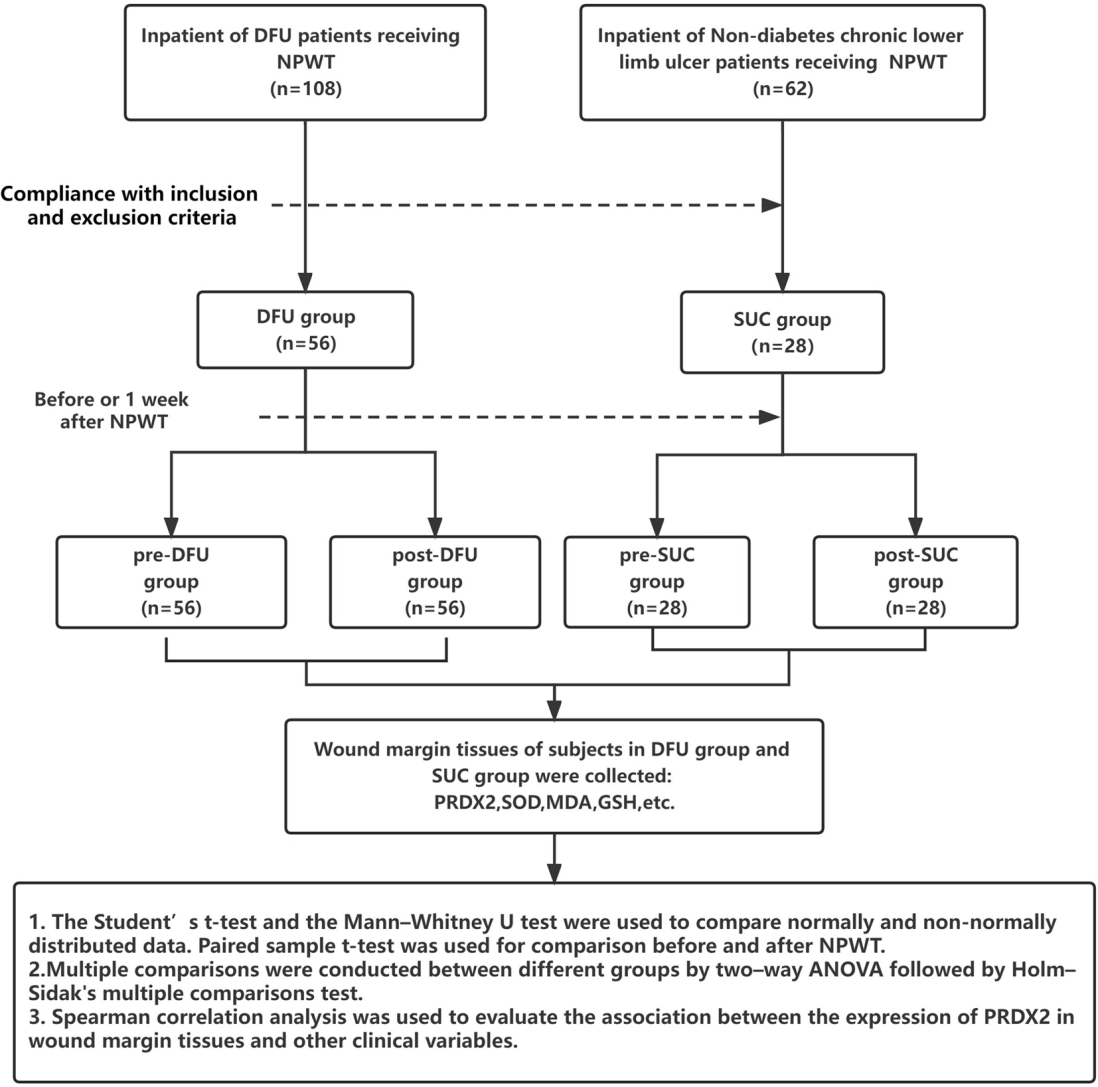
Clinical study design drawing. Abbreviations: DFU: diabetic foot ulcer; SUC: skin ulcer control; NPWT: negative pressure wound therapy; Pre-DFU: before NPWT in the DFU group; pre-SUC: before NPWT in the SUC group; PRDX2: peroxiredoxin-2; SOD: superoxide dismutase; MDA: malondialdehyde; GSH: glutathione.

A study was conducted on 108 patients with DFU who were admitted to the Department of Endocrinology at the First Affiliated Hospital of Anhui Medical University between June 2020 and June 2022 and underwent NPWT. According to the inclusion and exclusion criteria, 56 patients with DFU were ultimately included in the DFU group. On the basis of the NPWT timeline, the aforementioned 56 DFU patients were defined as the pre-DFU group before receiving NPWT, and as the post-DFU group one week after receiving NPWT. The inclusion criteria for all patients in the DFU group were as follows: (1) diagnosis of T2DM; (2) aged between 18 and 80 years; (3) ulcer duration of ≥ 4 weeks^25^; (4) ulcer area ranging from 2 to 20 cm^2^ with Wagner grade 2–3; (5) ankle-brachial index (ABI) ranging from 0.7 to 1.3. In addition, 62 non-diabetic patients with chronic lower limb skin ulcers who underwent NPWT at the Department of Burns during the same period were observed. According to the inclusion and exclusion criteria, a total of 28 patients were ultimately included in the skin ulcer control (SUC) group. In the same way, The above 28 patients in the SUC group were defined as the pre-SUC group before receiving NPWT, and as the post-SUC group one week after receiving NPWT. The inclusion criteria for all patients in the SUC group were as follows: (1) normal glucose tolerance; (2) age between 18 and 80 years; (3) absence of lower extremity arteriovenous lesions; (4) ulcer duration of ≥ 4 weeks; (5) ulcer area of 2–20 cm^2^; (6) ABI ranging from 1.0 to 1.3. Notably, participants with severe cardiac, liver, and kidney dysfunction; a history of malignant tumours and cancerous ulcer wounds; autoimmune disease; severe sepsis; and recent use of glucocorticoids, immunosuppressants, and exogenous cytokines within the last 6 months were excluded from the study. The research design is illustrated in Fig. 1. This study was approved by the medical ethics committee of the First Affiliated Hospital of Anhui Medical University as CDEC000004982, and informed consent was obtained from the subjects.

### Study methods

#### Collection and treatment of wound margin tissue

All participants received routine systemic treatment, including anti-infection, antihypertensive, lipid regulation, nerve nutrition, hypoproteinaemia improvement, and enhancement of lower limb wound blood supply. Additionally, patients in the DFU group received appropriate glycaemic control treatment. Both groups underwent wound debridement to remove blackened and necrotic soft tissue and bone tissue, followed by NPWT. Based on previous research reports^26^, the VAC^®^ negative pressure-assisted healing therapy system (KCI, USA) was used for NPWT, with the negative pressure set at 125 mmHg (1 mmHg = 0.133 kPa) for one week. Notably, in this study, polyurethane foam dressing was used to fill the wound. In principle, the type of foam dressings did not change during the period of one week. Additionally, NPWT used the same polyurethane foam dressing in both DFU and SUC groups. Before NPWT and one week after NPWT (upon removal of the negative pressure device), a skilled surgeon used tissue scissors to excide full-thickness skin tissue within a 0.5-cm range of the wound edge, according to the sampling plan. Wound healing in patients with DFU was evaluated after 4 weeks of treatment.

#### General data collection and laboratory index testing

Participants' sex and age were obtained through a questionnaire. The area of the wound ulcers was quantified using digital photography and ImageJ medical imaging software (Image J-IJ133-JDK15, National Institutes of Health). ABI was determined using a Doppler blood flow detector (DPL-03, Hangzhou Yuanxiang Medical, China).

After a minimum 8-h fasting period, venous blood samples were obtained from the median vein of the elbow using anticoagulant tubes containing sodium fluoride, EDTA, or heparin (the specific anticoagulant was selected based on the examination items) or non-anticoagulant collection tubes. The blood samples were obtained between 6:00 and 7:00 in the morning while the participants remained fasting. The collected samples were used to determine various indicators, including fasting plasma glucose (FPG), glycosylated haemoglobin A1c (HbA1c), white blood cell (WBC) count, neutrophil percentage (NEUT), C-reactive protein (CRP), and other indicators. FPG was measured using the glucose oxidase method, HbA1c was detected using high-pressure liquid chromatography, triglyceride (TG) and total cholesterol (TC) were measured using oxidase-linked colourimetry, and estimated glomerular filtration rate (eGFR) was used to evaluate renal function^27^. CRP and procalcitonin (PCT) were measured using latex-enhanced scattering immunoturbidimetry and immunoluminescence, respectively. The change values (△value) of the above observation indicators before and a week after NPWT were calculated using the formula: △ value = value after treatment − value before treatment.

#### Cell culture

Normal human dermal fibroblasts (NHDFs) were obtained from the National Cell Experimental Resource Bank of China. NHDFs were cultured in low-glucose Dulbecco's Modified Eagle Medium containing 10% foetal bovine serum (FBS) (Wisent Biotechnology Co., Ltd., Nanjing, China) and 1% penicillin/streptomycin (Yuanpei Biotechnology Co., Ltd., Shanghai, China). The cells were cultured at 37 °C under 5% carbon dioxide (CO_2_), and the medium was changed every 2–3 days. When the fusion degree of cell growth reached over 90%, as observed under microscopy, trypsin digestion and passage culture were performed. In subsequent experiments, three independent samples were used in each group.

#### Cell transfection and grouping

After cell passage, they were seeded onto a six-well plate and randomly divided into two groups, namely the NG control group (NG group, 5 mM D-glucose) and the HG intervention group (HG group, 50 mM D-glucose) for culture. PRDX2 siRNA was procured from RiboBio (Guangzhou, China). PRDX2 was amplified from a human complementary deoxyribonucleic acid (cDNA) template and cloned into the plasmid cloning DNA 3.1(+) vector (Invitrogen, USA). NHDFs were transfected with the plasmids using Lipofectamine 3000 (Invitrogen, USA) following the manufacturer’s protocol. The cells were harvested for subsequent analysis 24–48 h after transfection. The groups included: NG+si-NC (5 mM D-glucose control group transfected with PRDX2 small interfering ribonucleic acid [siRNA]), NG+si-PRDX2 (5 mM D-glucose experimental group transfected with PRDX2 siRNA), HG+si-NC (50 mM D-glucose control group transfected with PRDX2 siRNA), HG+si-PRDX2 (50 mM D-glucose experimental group transfected with PRDX2 siRNA); NG+OE-NC (5 mM D-glucose control group transfected with overexpressed PRDX2), NG+OE-PRDX2 (5 mM D-glucose experimental group transfected with overexpressed PRDX2), HG+OE-NC (50 mM D-glucose control group transfected with overexpressed PRDX2), HG+OE-PRDX2 (50 mM D-glucose experimental group transfected with overexpressed PRDX2).

#### Cell viability assay

Cell viability was measured using the Cell Counting Kit-8 (CCK-8) (Biosharp, Beijing, China). After normal digestion, the cells from each group were resuspended in a culture medium to a density of 1 × 10^5^ cells. A total of 100 µL of cell suspension was added to each well of a 96-well plate, and the marginal wells were filled with sterile phosphate-buffered saline (PBS). The edge holes were filled with sterile PBS. The inoculated cell culture plate was incubated overnight under 5% CO_2_ at 37 °C. After 48 h of incubation, 10 µL of CCK-8 reagent was added to each well. The absorbance values of each well were measured at an optical density of 450 nm using an enzyme-linked immunosorbent assay.

#### Wound healing assay

Cells in the logarithmic growth stage were seeded in a six-well plate at a density of 1 × 10^6^ cells/well, and three holes were set in each group. Once the cell monoliths had adhered to the wall, the six-well plate was scraped vertically using a 200 µL pipetting tip to avoid tilting. The suspended cells were cleaned thrice with PBS, the scratched cells were removed, and the cells were removed by adding serum-free medium and cultured in an incubator at 37 °C under 5% CO_2_. Photographs were obtained under microscopy at 0 h and 24 h respectively, and the experiment was repeated thrice.

#### Transwell migration assay

Transwell analysis was used to assess the NHDF migration ability. The transwell migration assay was conducted using 24-well hanging inserts (A200050, Millipore, Germany). Briefly, 100 µL of cell suspension was added to the upper cavity of a transwell insert and pore size was 8 µm, while 600–800 µL of medium containing 10% FBS was added to the lower cavity and cultured at 37 °C for 24 h. After incubation, the transwell chamber was removed from the incubator, and the medium was discarded. The chamber was washed twice with PBS, followed by fixation at room temperature with 1 mL 4% paraformaldehyde for 20 min. The chamber was then air dried, and staining was performed using 1 mL of 0.1% crystal violet at room temperature for 20 min. After gently washing with clean water and several rinses, the chamber was removed, and the liquid in the upper chamber was absorbed. Subsequently, carefully discarded ungrounded cells from the surface of the upper chamber using a wet cotton swab, followed by washing with PBS three times. Three random fields were selected for cell counting, and the number of cells that migrated to the lower insert was calculated using ImageJ.

#### Cell apoptosis assay

Cell apoptosis was assessed using the annexin V-fluorescein isothiocyanate (FITC)/propidium iodide (PI) apoptosis detection kit (Solarbio, Beijing, China). In brief, NHDFs were resuspended in 100 μL of Binding Buffer and stained with annexin V-FITC (5 μL) and PI (5 μL) for 15 min in a dark environment. Flow cytometry was then performed, and the apoptosis images were analysed using NovoExpress software.

#### Western blot

PRDX2 expression in the wound margin tissue (T-PRDX2) was analysed using Western blotting. Frozen wound margin tissue and NHDFs were lysed using RIPA lysis buffer (Biosharp, Beijing, China). The proteins were isolated using SDS-PAGE and transferred onto PVDF membranes. After sealing with 5% skimmed milk powder, the membranes were incubated overnight at 4 °C with with a 1:1000 dilution of primary antibody rabbit anti-PRDX2 (DF6691, Affinity, China) and mouse anti-β-actin (TA-09, Zs-BIO, China). Subsequently, the membranes were incubated with horseradish peroxidase (HRP)-conjugated secondary antibodies (ab205718, Abcam, UK) at room temperature for 2 h. Signal detection was performed using ECL substrates, and the grayscale values of target proteins and internal reference grayscale ratios (β-actin) were detected using ImageJ software for analysis.

#### Quantitative-reverse transcription polymerase chain reaction (qRT-PCR) analysis

qRT-PCR was used to detect the PRDX2 expression in NHDFs. Total RNA was extracted using TRIzol reagent (Thermo Fisher, Shanghai, China), and cDNA was synthesised using PrimeScript RT kit (TaKaRa, Beijing, China). PRDX2 messenger RNA was analysed using SYBR™ Green PCR Master Mix (Servicebio, Wuhan, China). The relative PRDX2 levels were calculated using the 2^−△△Ct^ method, with β-actin as the internal reference. The primers were as follows:

- PRDX2: forward primer 5′-AAAGAGGTGAAGCTGTCGGA-3′ and reverse primer 5′-CAGGTGGGTGAACTGAGAGT-3′;
- β-actin: forward primer 5′-CCCTGGAGAAGAGCTACGAG-3′ and reverse primer 5'-GGAAGGAAGGCTGGAAGAGT-3′.

#### Determination of oxidative stress markers

The tissue to be measured was accurately weighed, add nine times the volume of normal saline was added based on the ratio of mass (g) to volume (mL) of 1:9. The tissue was thoroughly ground using an electric grinder, followed by centrifugation in a frozen centrifuge (2500 r/min, 4 °C) for 10 min. The supernatant was collected and transfected to a 2 mL centrifuge tube for further analysis. The average fluorescence intensity of ROS, the activity of superoxide dismutase (SOD) and the contents of malondialdehyde (MDA) and reduced glutathione (GSH) were determined using relevant kits. The mean fluorescence intensity of ROS cells was measured using the dichloro-dihydro-fluorescein diacetate assay. SOD activity was determined using a water-soluble tetrazole assay. MDA content was measured using the thiobarbituric acid assay, and the content of GSH was determined using the dithiodinitrobenzoic acid assay.

### Statistical analysis

Data analysis was conducted using SPSS 22.0 (Chicago, IL, USA). Normally distributed measurement data are expressed as the mean ± standard deviation, and non-normally distributed measurement data are expressed as the median (interquartile range) [M (P25, P75)]. Student’s t-test and the Mann–Whitney U test were used to compare normally and non-normally distributed data. Paired sample t-test was used for comparison before and after NPWT. Multiple comparisons were conducted between different groups by two‐way ANOVA followed by Holm–Sidak's multiple comparisons test. Enumeration data are presented as a percentage and analysed using the χ^2^ test. Spearman’s correlation analysis was used to evaluate the association between T- PRDX2 and other clinical variables. Differences were considered statistically significant with a *P* < 0.05.

### Ethics approval and consent to participate

All procedures performed in this study involving human participants were in accordance with the 1964 Helsinki Declaration and its later amendments or comparable ethical standards. This study was approved by the medical ethics committee of the First Affiliated Hospital of Anhui Medical University as CDEC000004982, and informed consent was obtained from the subjects.

## Results

### Comparison in baseline data between the DFU and SUC groups

No differences were observed in terms of sex, age, ulcer duration, and ulcer area between the two groups (*P* > 0.05). As presented in Table 1, FPG, HbA1c, TG, and TC of the SUC group were significantly lower (*P* < 0.05), while body mass index (BMI), ABI, albumin (ALB), and eGFR were significantly higher (*P* < 0.05) than those of the DFU group.

**Table 1:** Comparison in baseline data between DFU group and SUC group [($\overline{x} \pm s$), M (P25, P75)].

Variable	DFU (n = 56)	SUC (n = 28)	*P* value
Sex (male/famale)	56 (38/18)	28 (20/8)	> 0.05
Age (year)	60.15 ± 10.87	59.94 ± 13.03	> 0.05
BMI (kg/m^2^)	22.46 ± 2.07	24.26 ± 2.59	< 0.05
Ulcer duration (week)	6.83 ± 0.97	6.50 ± 1.26	> 0.05
Ulcer area (cm^2^)	10.15 ± 1.72	10.59 ± 2.43	> 0.05
ABI	0.82 ± 0.06	1.16 ± 0.07	< 0.05
FPG (mmol/L)	10.27 ± 3.25	5.12 ± 0.45	< 0.05
HbA1c (%)	9.93 ± 2.45	5.16 ± 0.24	< 0.05
ALB(g/L)	35.26 ± 2.72	40.26 ± 1.60	< 0.05
TG (mmol/L)	1.23 (0.94,1.51)	0.94 ± 0.17	< 0.05
TC (mmol/L)	3.53 ± 0.98	2.94 ± 0.15	< 0.05
eGFR (ml/min/1.73m^2^)	82.77 ± 12.54	101.56 ± 16.47	< 0.05

Data are presented mean ± standard deviations or numbers (%) or median with IQR. Differences between two groups analyzed using t test or nonparametric test (Mann Whitney U).

*DFU* diabetic foot ulcer group, *SUC* skin ulcer control group; *BMI* body mass index, *ABI* ankle brachial index, *FPG* fasting plasma glucose, *HbA1c* glycated hemoglobin A1c, *ALB* albumin, *TG* triacylglycerol, *TC* total cholesterol, *eGFR* estimated glomerular filtration rate.

### Comparison of primary parameters between the DFU and SUC groups before and a week after NPWT

As presented in Table 2, significant differences were observed between the pre-SUC group and the pre-DFU group. The inflammatory indicators (WBC, NEUT, CRP, and PCT) and MDA in the pre-DFU group were significantly higher in the pre-SUC group (*P* < 0.05), while SOD, GSH, and T-PRDX2 were significantly lower (*P* < 0.05). In the DFU and SUC groups, after a week of NPWT, there were significant decreases in inflammatory indicators (WBC, NEUT, CRP, and PCT) and MDA levels compared with before NPWT (*P* < 0.05). Conversely, there were significant increases in SOD, GSH, and T-PRDX2 levels (*P* < 0.05).

**Table 2:** Comparison of primary parameters between before NPWT and a week after NPWT in the DFU group and SUC group [($\overline{x} \pm s$), M (P25, P75)].

Variable	DFU(n = 56)	SUC(n = 28)	*P* value
WBC (*10^9/L)			
Before NPWT	17.86 ± 2.47	11.43 ± 2.12	< 0.05
After NPWT	8.08 ± 1.32^a^	7.84 ± 1.26^a^	–
NEUT (%)			
Before NPWT	77.07 ± 13.66	72.40 ± 7.02	< 0.05
After NPWT	69.85 ± 10.62^a^	67.11 ± 5.23^a^	–
CRP (mg/L)			
Before NPWT	59.07 ± 3.61	26.97 ± 4.77	< 0.05
After NPWT	14.50 (7.18, 43.95)^a^	13.77 ± 2.68^a^	–
PCT (ng/ml)			
Before NPWT	2.96 ± 0.52	1.78 ± 0.27	< 0.05
After NPWT	0.54 ± 0.07^a^	0.15 ± 0.04^a^	–
SOD (U/mg)			
Before NPWT	31.34 ± 4.52	34.99 ± 4.13	< 0.05
After NPWT	41.67 ± 2.92^a^	42.68 ± 2.93^a^	–
GSH (umol/g)			
Before NPWT	259.12 ± 30.52	302.10 ± 22.57	< 0.05
After NPWT	396.19 ± 26.75^a^	420.07 ± 16.15^a^	–
MDA (nmol/mg)			
Before NPWT	2.89 ± 0.19	2.63 ± 0.33	< 0.05
After NPWT	1.83 ± 0.45^a^	1.68 ± 0.29^a^	–
T-PRDX2			
Before NPWT	0.45 ± 0.07	0.60 ± 0.06	< 0.05
After NPWT	0.67 ± 0.06^a^	0.80 ± 0.07^a^	–

Data presented as mean ± standard deviation or median with interquartile range (IQR). Multiple comparisons were conducted between different groups by two‐way ANOVA followed by Holm–Sidak's multiple comparisons test versus before NPWT in each group, ^a^*P* < 0.05.

*NPWT* negative pressure wound therapy, *DFU* diabetic foot ulcer group, *SUC* skin ulcer control group, *WBC* white blood cell, *NEUT* percentage of neutrophils, *CRP* C-reactive protein, *PCT* pro calcitonin, *SOD* superoxide dismutase, *GSH* glutathione, *MDA* malondialdehyde, *T-PRDX2* peroxiredoxin-2 protein expression in wound margin tissue.

### Correlations between T-PRDX2 and other clinical data in the DFU and SUC groups before NPWT

**Figure 2:**
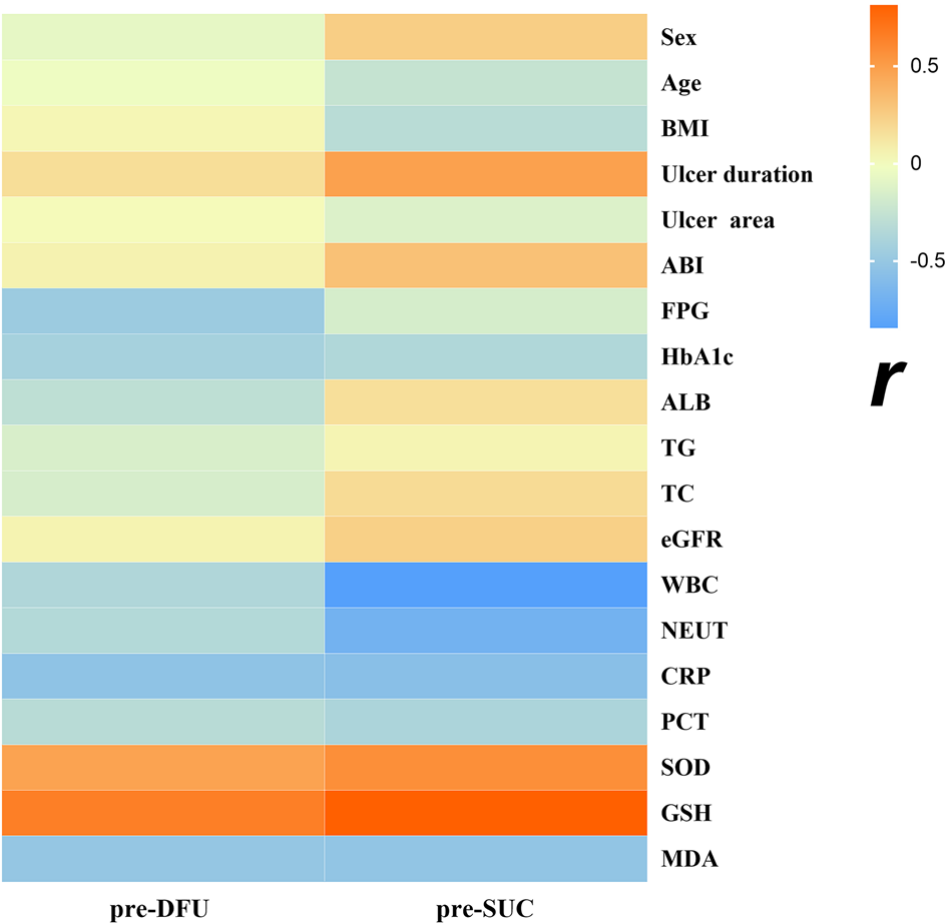
Correlations between T-PRDX2 expression levels and other clinical data in the DFU and SUC groups before NPWT (r). Abbreviations: Pre-DFU: before NPWT in the diabetic foot ulcer group; pre-SUC: before NPWT in the skin ulcer control group; BMI: body mass index; ABI: ankle brachial index; FPG: fasting plasma glucose; HbA1c: glycated hemoglobin A1c; ALB: albumin; TG: triacylglycerol; TC: total cholesterol; eGFR: estimated glomerular filtration rate; WBC: white blood cell; NEUT: neutrophil percentage; CRP: C-reactive protein; PCT: Pro Calcitonin; SOD: superoxide dismutase; GSH: glutathione; MDA: malondialdehyde.

As presented in Fig. 2, in the pre-DFU group, there was a negative correlation between the T- PRDX2 expression level and FPG, HbA1c, and ALB (*P* < 0.05). Similarly, in the pre-DFU and pre-SUC groups, the T-PRDX2 expression level was negatively correlated with inflammatory indicators (WBC, NEUT, CRP, and PCT) and MDA (*P* < 0.05), and a positive correlation was observed with SOD and GSH (*P* < 0.05). However, there was no significant correlation observed with other clinical parameters (*P* > 0.05).

### Comparison of △ values of primary parameters between the DFU and SUC group before and a week after NPWT

As presented in Table 3, the changes in the inflammatory indicators (△ WBC, △ NEUT, △ CRP, and △ PCT) before and after NPWT were significantly higher in the DFU group compared with the SUC group (*P* < 0.05). Similarly, the changes in oxidative stress markers (△ SOD, △ GSH, and △ MDA) in the wound margin tissue also exhibited significant differences between the two groups (*P* < 0.05). Particularly noteworthy is the observation that △ T-PRDX2 in the DFU group were significantly higher than those in the SUC group (*P* < 0.05).

**Table 3:** Comparisons of ∆ value of primary parameters before and after NPWT between DFU group and SUC group [($\overline{x} \pm s$), M (P25, P75)].

Variable	DFU	SUC	*P* value
△WBC (*10^9/L)	− 4.70 (− 9.98, − 3.05)	− 3.59 ± 0.49	< 0.05
△NEUT (%)	− 7.53 ± 1.46	− 5.30 ± 0.89	< 0.05
△CRP (mg/L)	− 36.60 ± 3.91	− 13.20 ± 1.84	< 0.05
△PCT (ng/ml)	− 2.39 ± 0.27	− 1.62 ± 0.30	< 0.05
△SOD (U/mg)	11.19 ± 2.17	8.93(7.56,13.83)	< 0.05
△GSH (μmol/g)	141.29(97.86,193.33)	117.97 ± 18.78	< 0.05
△MDA (nmol/mg)	− 1.02(− 1.22, − 0.96)	− 0.96(− 1.02, − 0.89)	< 0.05
△T-PRDX2	0.22 ± 0.03	0.20 ± 0.02	< 0.05

Data presented as mean ± standard deviation or median with interquartile range (IQR). Differences between two groups analyzed using t test or nonparametric test (Mann Whitney U).

*DFU* diabetic foot ulcer group, *SUC* skin ulcer control group, *WBC* white blood cell, *NEUT* percentage of neutrophils, *CRP* C-reactive protein, *PCT* pro calcitonin, *SOD* superoxide dismutase, *GSH* glutathione, *MDA* malondialdehyde, *T-PRDX2* peroxiredoxin-2 protein expression in wound margin tissue. △: the change value of various indexes before and after treatment.

### Relationship between △ T-PRDX2 and other clinical features of skin ulcers in the DFU and SUC groups

The median values of △ T-PRDX2 in the DFU and SUC groups were used as the cut-off values to further investigate the clinical significance of the change in the PRDX2 expression level in wound margin tissue after NPWT. Participants with values below the median were classified as the T-PRDX2 low change group, while those with values equal to or greater than the median were classified as the T-PRDX2 high change group. As presented in Table 4, it was observed that in the DFU and SUC groups, △ T-PRDX2 was positively correlated with the wound healing rate 4 weeks after treatment (DFU group *P* = 0.004, SUC group *P* = 0.031). Additionally, after 4 weeks of stopping NPWT, the rate of complete wound healing was 41.1% (23/56) in the DFU group and 64.3% (18/28) in the SUC group. The healing rate in the SUC group was significantly higher than that in the DFU group (chi-square = 4.026, *P* = 0.045).

**Table 4:** Relationship between ∆T-PRDX2 and clinical features of skin ulcers in DFU group and SUC group[($\overline{x} \pm s$), M (P25, P75)].

	DFU			SUC		
	High change group (n = 34)	Low change group (n = 22)	*P* value	High change group (n = 16)	Low change group (n = 12)	*P* value
Age (year)	59.83 ± 9.25	60.94 ± 10.77	0.211	59.23 ± 9.08	60.08 ± 10.23	0.198
Sex			0.890			0.729
Male	22(64.7)	16(72.7)		11(68.8)	9(75.0)	
Female	12(35.3)	6(27.3)		5(31.2)	3(25.0)	
Ulcer duration (week)			0.943			0.733
≤ 6	8(23.5)	5(22.7)		5(31.2)	4(33.3)	
6–10	16(47.1)	11(50.0)		7(43.8)	6(50.0)	
> 10	10(29.4)	6(27.3)		4(25.0)	2(16.7)	
Ulcer area (cm^2^)			0.887			0.885
≤ 5	11(32.3)	7(31.8)		4(25.0)	3(25.0)	
5–10	14(41.2)	10(45.5)		6(37.5)	5(41.7)	
> 10	9(26.5)	5(22.7)		6(37.5)	4(33.3)	
Wagner			0.725			
II	14(41.2)	8(36.4)		–	–	
III	20(58.8)	14(63.6)		–	–	
Ulcer healing rate after 4 weeks (%)			0.004			0.031
Healing	19(55.9)	4(18.2)		13(81.2)	5(41.7)	
Non-healing	15(44.1)	18(81.8)		3(18.8)	7(58.3)	

Data are presented mean ± standard deviation or numbers (%); differences between two groups analyzed using t-test or χ^2^ test.

*DFU* diabetic foot ulcer group, *SUC* skin ulcer control group.

### The effect of PRDX2 on NHDF function

**Figure 3:**
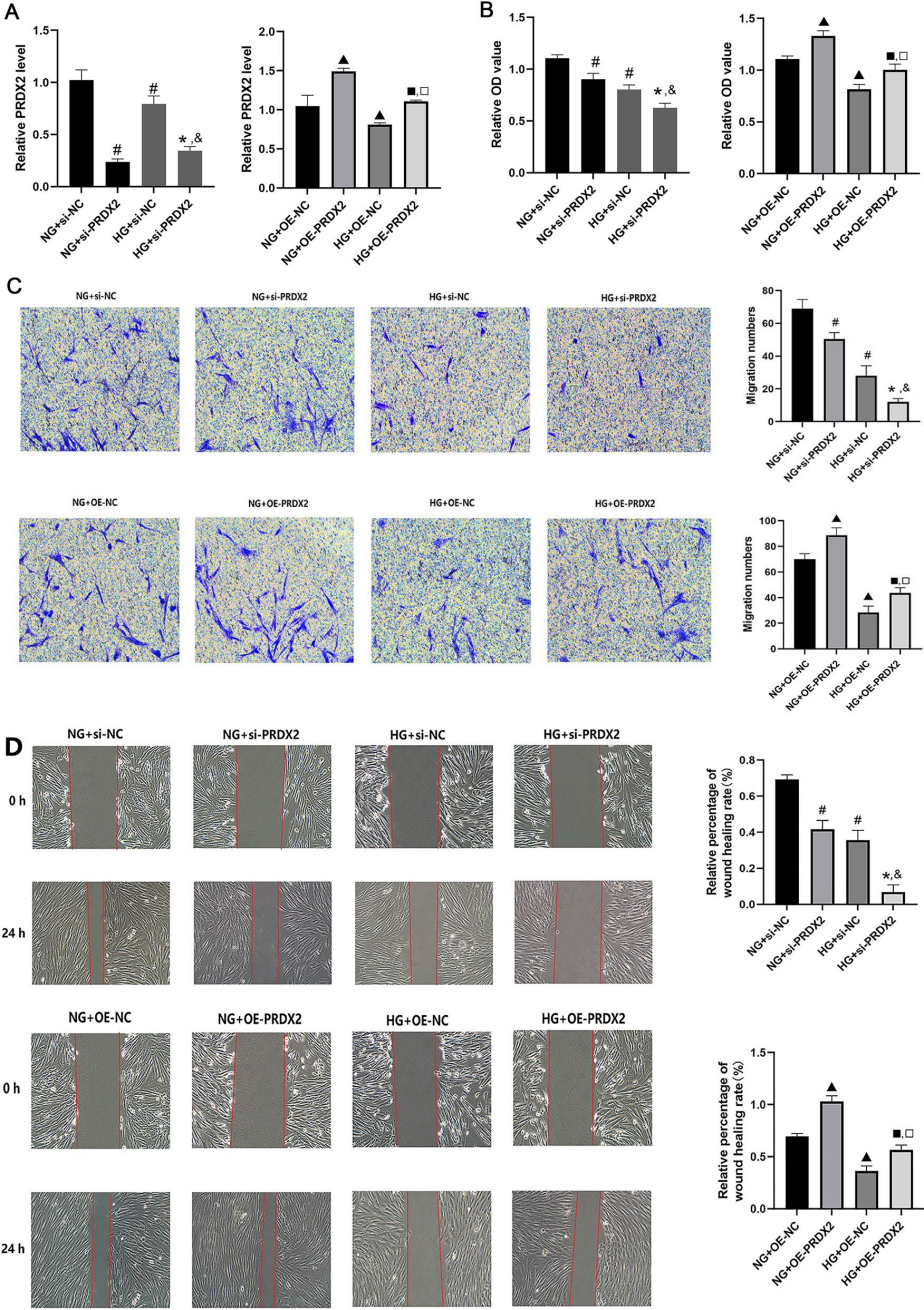

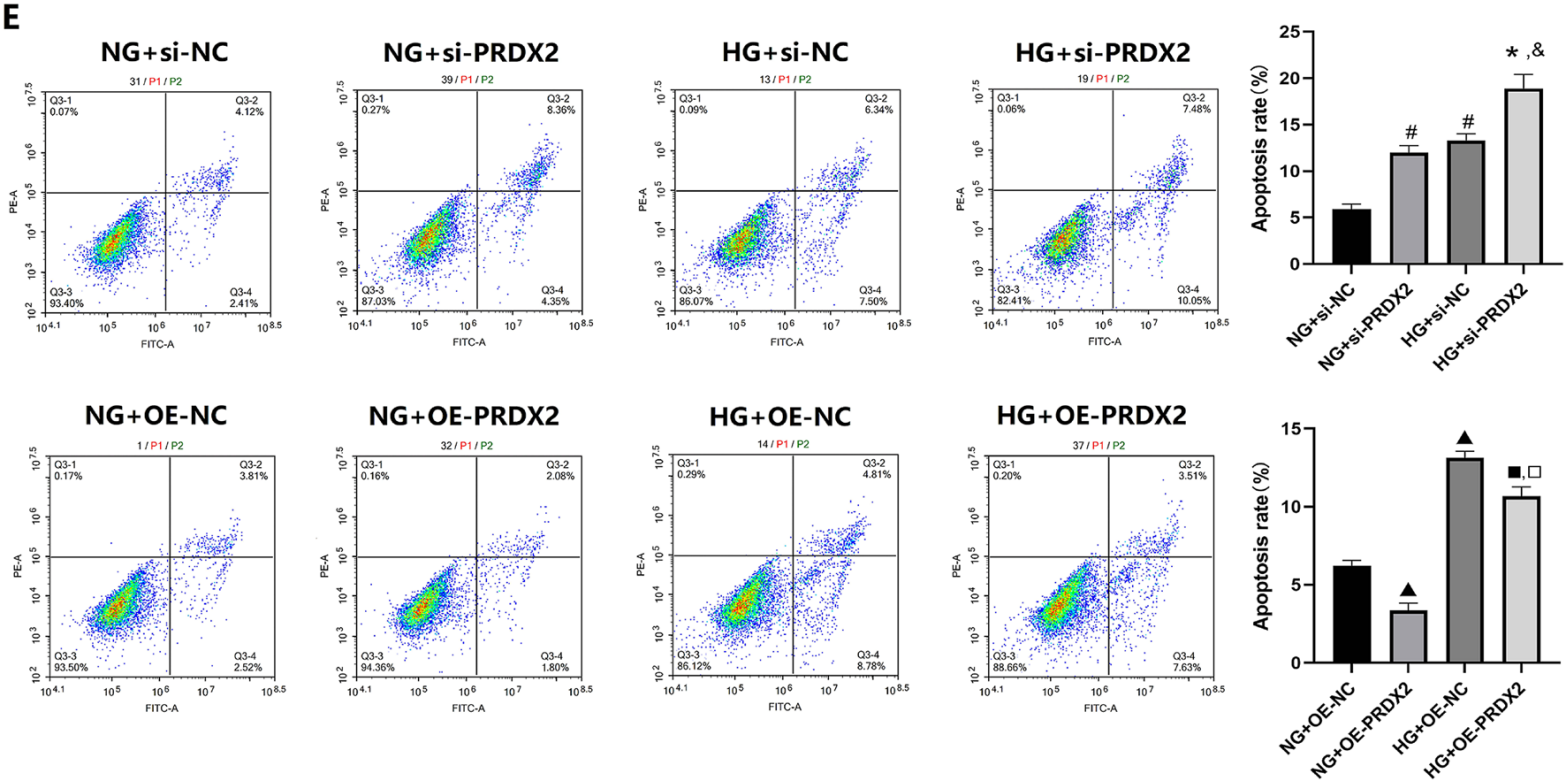
Effect of PRDX2 on proliferation, migration, and apoptosis of NHDF cells. (**A**) The mRNA expression levels of PRDX2 by qRT-PCR in NHDF cell under different conditions. (**B**) Cell proliferation was monitored by CCK8 assay. (**C** and **D**) Cell migratory capability was measured by Transwell assay and wound healing assay. (**E**) Cell apoptosis was measured by flow cytometry (Annexin V-PI); versus NG+Si-NC, ^#^*P* < 0.05; versus HG+Si-NC, ^*^P < 0.05; versus NG+si-PRDX2, ^&^*P* < 0.05; versus NG+OE-NC, ^▲^*P* < 0.05; versus HG+OE-NC, ^■^*P* < 0.05; versus NG+OE-PRDX2, ^□^*P* < 0.05.

To investigate the effect of PRDX2 on NHDF function the following experiment was conducted: PRDX2 content was measured using qRT-PCR. The findings revealed a significant decrease in PRDX2 expression in NHDFs transfected with PRDX2 siRNA under NG and HG culture conditions compared with the NC group (*P* < 0.05). Conversely, NDHFs transfected with the PRDX2 overexpression plasmid exhibited a significant increase in PRDX2 expression under NG and HG culture conditions compared with the NC group (*P* < 0.05) (Fig. 3A), indicating successful transfection.

CCK-8 assay was performed further clarify the effect of PRDX2 on NHDF activity. The findings demonstrated that cell viability was significantly decreased under HG culture conditions compared with NG culture conditions (*P* < 0.05). Additionally, si-PRDX2 transfection significantly reduced cell viability under NG and HG culture conditions (*P* < 0.05), whereas PRDX2 overexpression significantly enhanced cell viability (*P* < 0.05) (Fig. 3B).

The transwell migration and wound healing assays were conducted to determine the effect of PRDX2 on NHDF cell migration. The transwell assay revealed that cell migration was significantly inhibited under HG culture conditions compared with NG culture conditions (*P* < 0.05). Furthermore, si-PRDX2 transfection significantly inhibited NHDF migration under NG and HG conditions, while PRDX2 overexpression significantly promoted cell migration (*P* < 0.05) (Fig. 3C). The wound healing assay demonstrated that NHDFs transfected with PRDX2 siRNA exhibited a significantly lower migration rate than the NC group under NG and HG culture conditions (*P* < 0.05). Conversely, PRDX2 overexpression significantly increased the cell migration rate (*P* < 0.05) (Fig. 3D).

Flow cytometry analysis was performed to assess NHDF apoptosis, revealing that HG culture conditions significantly increased the apoptosis rate compared with NG conditions. Moreover, NHDFs transfected with PRDX2 siRNA exhibited a significant increase in apoptosis rate under NG and HG conditions (*P* < 0.05), while PRDX2 overexpression significantly reduced the NHDF apoptosis rate (*P* < 0.05) (Fig. 3E). In conclusion, HG culture conditions inflicted certain damage to NHDF function, while PRDX2 demonstrated the ability to enhance NHDF vitality and migration, along with reducing the rate of cell apoptosis.

### Changes in oxidative stress markers in NHDFs under HG conditions

**Figure 4:**
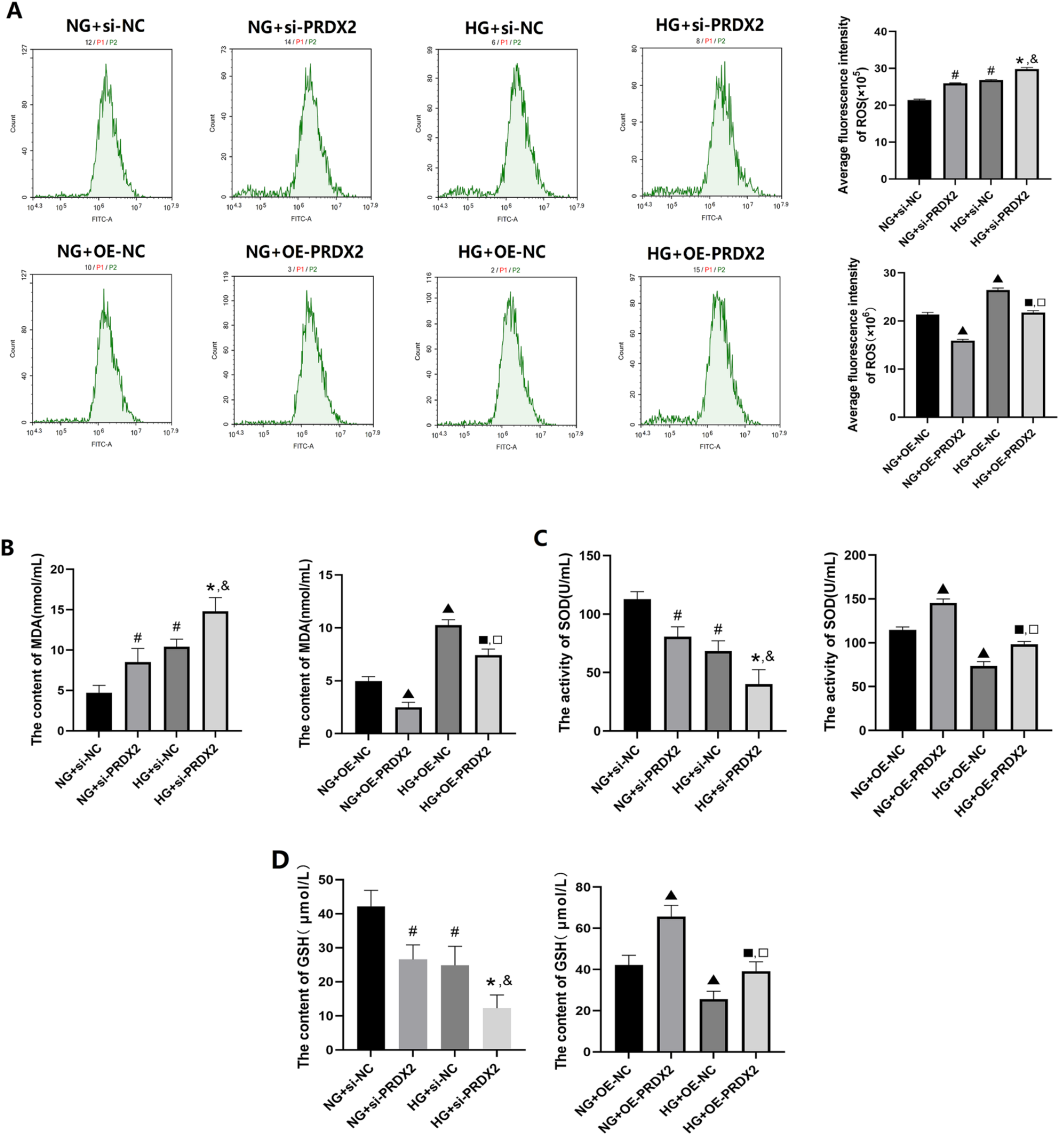
The effect of PRDX2 on oxidative stress markers in NHDF cells. (**A**) The average fluorescence intensity of ROS was detected by flow cytometry. (**B**) The content of MDA was detected by biochemical method. (**C**) The activity of SOD was detected by biochemical method. (**D**) The content of GSH was detected by biochemical method. versus NG+Si-NC, ^#^*P* < 0.05; versus HG+Si-NC, ^*^P < 0.05; versus NG+si-PRDX2, ^&^*P* < 0.05; versus NG+OE-NC, ^▲^*P* < 0.05; versus HG+OE-NC, ^■^*P* < 0.05; versus NG+OE-PRDX2, ^□^*P* < 0.05.

As presented in Fig. 4, the relevant reagent kits were used to identify the oxidative stress markers in NHDFs. The findings demonstrated that HG culture conditions significantly increased the ROS and MDA levels compared with NG culture conditions (*P* < 0.05), while simultaneously decreasing GSH and SOD levels (*P* < 0.05). In addition, compared with NHDFs treated in the NC group, NHDFs transfected with si-PRDX2 exhibited a significant increase in ROS and MDA levels (*P* < 0.05) and a significant decrease in GSH and SOD levels (*P* < 0.05) under NG and HG culture conditions. Conversely, irrespective of NG or HG conditions, PRDX2 overexpression could result in decreased ROS and MDA levels in NHDFs (*P* < 0.05) and increased GSH and SOD levels (*P* < 0.05). These findings indicate that HG conditions could enhance oxidative stress in NHDFs, while PRDX2 plays a role in mitigating oxidative stress in NHDFs.

## Discussion

Foot ulcers and infections are common among individuals with diabetic foot. Individuals with diabetes often experience impaired leukocyte chemotaxis and phagocytosis, resulting in a 17-fold increased risk of wound infection. This can lead to progressive damage of the soft tissues and bones of patients with DFU, significantly increasing the risk of amputation^28^. A recent meta-analysis, which included 27 studies, revealed a higher overall mortality rate in DFU, with a 5-year mortality rate of approximately 50%, with infection being the primary cause of death^29^. This study observed significantly higher levels of inflammatory indicators (WBC, NEUT, CRP, and PCT) in patients with DFUs. Similarly, non-diabetic patients with chronic lower limb ulcers also exhibited higher levels of inflammatory indicators beyond normal values. These findings might reflect the common characteristics of chronic wounds, such as persistent infection, inadequate tissue perfusion or hypoxia, and impaired cellular function. These factors contribute to a prolonged inflammatory phase and serve as the pathological basis for delayed wound healing^30^. Previous studies have indicated that diabetes wounds exhibit increased inflammatory cell infiltration and increased oxidative stress compared with normal wounds^31^. Furthermore, recent investigations have identified a link between the development of diabetic foot and the exacerbation of oxidative stress and inflammatory processes^32^. The findings of this study revealed significantly higher levels of inflammatory indicators in patients with DFU compared to those with non-diabetic chronic lower limb ulcers. Additionally, DFUs exhibited a more pronounced oxidative stress response compared with non-diabetic chronic lower limb ulcer wounds. When considering the wound healing rate of all participants, these findings suggest that the strong oxidative stress and inflammatory response in diabetes wounds contribute to the increased difficulty in healing compared with ordinary wounds^33^.

Due to its negative pressure, drainage capabilities, semipermeable membrane, and dressing characteristics, NPWT has been demonstrated to inhibit bacterial growth, prevent cross-infection, and enhance the bactericidal effects of phagocytes and white blood cells, thereby accelerating wound closure^34^. Animal experiments have demonstrated that NPWT reduces bacterial counts^35^. Moreover, NPWT has been proven effective in treating cardiac pacemaker pocket infections^36^. A retrospective analysis study reported that NPWT significantly improved wound healing rates, shortened wound healing time, increased daily wound healing area, decreased hospital stay, and reduced adverse events in patients with surgical site infections^37^. Similarly, another study also confirmed that NPWT can reduce inflammatory reaction, increase the postoperative healing ratio of foot wounds and ulcers in diabetes patients, and shorten the wound healing time^38^. Consistent with previous research, our study found a significant decrease in inflammatory indicators after NPWT in the DFU and SUC groups. Human wound tissue experiments have demonstrated increased SOD activity in wound tissue after NPWT, and the application of SOD mimetics as local drugs in animal models promoted wound healing^39^. Additionally, other studies have revealed that wound tissues treated with NPWT exhibited a significant decrease in MDA and decreased cell damage caused by oxidative stress, leading to improved wound healing compared with the control group^40^. In agreement with these findings, our study observed that after NPWT, the levels of MDA in wound tissue of DFU and SUC groups were significantly reduced, while the levels of GSH and SOD were significantly increased. Collectively, NPWT can promote chronic wound healing by alleviating inflammatory reactions and oxidative stress.

PRDX2 is an important antioxidant enzyme that plays various biological roles by regulating different signalling pathways and mitigating oxidative stress reactions in physiological or pathological conditions. It also affects cell proliferation and differentiation and plays a role in the onset and progression of related diseases through its chaperone activity. In our study, a negative correlation was observed between the T-PRDX2 expression level and inflammatory markers and MDA, while a positive correlation was observed with SOD and GSH in the pre-DFU and pre-SUC groups. These findings suggest that PRDX2, a novel antioxidant, might participate in the oxidative stress process of chronic wounds and exert anti-inflammatory and antioxidant effects. An animal experiment demonstrated that PRDX2 knockout mice are more susceptible to lipid accumulation compared with the control group^41^. Furthermore, a previous in vitro study confirmed that shPRDX2 triggers adipocyte death and impairs adipocyte function through ROS production^42^. Besides, studies have reported that tripterine inhibits PRDX2, leading to increased intracellular ROS levels, which induces ROS-dependent endoplasmic reticulum stress, mitochondrial dysfunction, and apoptosis in gastric cancer cells^43^. Previous studies have demonstrated that long-term standardised physical exercise could increase PRDX2 levels in red blood cells of patients with T2DM, thereby reducing oxidative stress reactions and delaying disease progression^44^. Moreover, a study has demonstrated that increased serum PRDX2 concentration in patients with acute myocardial infarction exerts a protective effect on cells under hypoxic and inflammatory conditions, mitigating oxidative stress^45^. Additionally, it has been observed that tripterine promotes ROS production by inhibiting PRDX2, leading to the deironisation of activated hepatic stellate cells and exhibiting an anti-fibrotic effect^46^. In our study, a negative correlation was observed between the T-PRDX2 expression level in the pre-DFU group and FPG, HbA1c and MDA. This suggests that higher glucose concentrations and more pronounced oxidative stress responses are associated with lower levels of the antioxidant PRDX2. However, the role of PRDX2 in promoting wound healing of DFU with NPWT has rarely been reported. As described in this study, PRDX2 expression was significantly increased in wound margin tissue after a week of NPWT in the DFU and SUC groups, with a more pronounced change observed in the DFU group. Similarly, the changes in inflammatory indicators and oxidative stress markers before and after NPWT were more pronounced in the DFU group, which might be associated with the strong inflammatory response and intense oxidative stress caused by hyperglycaemia^31^. Subsequent correlation analysis revealed a positive correlation between increased PRDX2 expression after NPWT and the 4-week wound healing rate in the DFU and SUC groups. In other words, a larger increase of PRDX2 expression level was associated with a higher rate of wound healing. To the best of our knowledge, this is the first study to explore the association between T-PRDX2 changes in patients with DFU before and after NPWT and the outcomes of DFU treatment.

It is noteworthy that the T-PRDX2 expression level in the DFU group was significantly lower than that in the SUC group, with both groups comprising participants matched for sex and age. In vitro experiments were conducted using NHDFs to further reveal the changes in PRDX2 expression during wound healing under different glucose environments, considering the crucial role of fibroblasts in wound healing. The results of these experiments confirmed that under HG conditions, the baseline expression level of PRDX2 in NHDFs was lower compared with low glucose conditions. The SOD and GSH expression levels at different glucose concentrations were consistent with PRDX2, while the ROS and MDA expression levels exhibited the opposite trend. These findings align with the results obtained from clinical specimens. Additionally, it was observed that the si-PRDX2 group exhibited significantly increased ROS and MDA levels, along with significantly decreased GSH and SOD levels under NG and HG environments compared with the NC group. Conversely, the overexpressed PRDX2 group demonstrated contrasting results. These outcomes suggest that PRDX2, functioning as an antioxidant, plays a crucial role in counteracting oxidative stress in NHDFs. Moreover, this study highlights that oxidative stress markers still exhibited significant differences in the si-PRDX2 and OE-PRDX2 groups under varying glucose concentrations, further underscoring the heightened oxidative stress response induced by elevated glucose levels in fibroblasts.

Prior research has demonstrated a positive correlation between increased PRDX2 expression and the proliferation ability of spermatid cells^47^. Feng et al. reported that PRDX2 knockdown significantly inhibited the migration, invasion, and cell cycle progression of oesophageal cancer cells^48^. Zhang et al. reported that PRDX2 is highly expressed in gastric cancer cells, promoting cell proliferation and inhibiting apoptosis^49^. Other studies have revealed that PRDX2 gene knockout inhibits the growth of colorectal cancer cells, potentially mediated by the p38/FOXO pathway, which regulates cell cycle and autophagy, thus indicating a role for PRDX2 in promoting cell proliferation^50^. In our study, it was observed that silencing PRDX2 suppressed the proliferation and wound-healing abilities of NHDFs under different glucose environments while promoting cell apoptosis. Notably, compared to NG environments PRDX2-silenced NHDFs exhibited lower proliferation and wound healing abilities, along with higher cell apoptosis rates under HG environments. Therefore, the regulatory effect of PRDX2 on cell function was further validated by overexpressing PRDX2 in NHDFs. It was observed that PRDX2 overexpression could promote the proliferation and wound-healing ability of NHDFs under different glucose environments while reducing the rate of cell apoptosis.In summary, the findings suggest that PRDX2 overexpression promoted the proliferation and migration of NHDFs under different glucose environments while reducing the rate of cell apoptosis. These mechanisms might promote chronic wound healing by NPWT, with PRDX2 playing a significant role in this process.

This study has several limitations. First, this study is a single-centre sample study, and the limited clinical sample size might have led to slight selection bias. Second, in the clinical setting, the absence of an established control group receiving a common treatment makes it challenging to accurately assess whether the observed changes in PRDX2 expression in chronic wound tissue are solely attributed to the unique NPWT intervention. Third, the findings of this study are based solely on biochemical and functional studies using in vitro two-dimensional single culture systems. The translation of these results to clinical treatments remains limited. Finally, although the cell lines used in this study are from the National Cell Experimental Resource Bank of China, they are derived from neonatal foreskin fibroblasts, which may have certain limitations in the study of adult wound fibroblasts. Therefore, further in vitro and in vivo studies are necessary to validate our findings and evaluate the potential of PRDX2 as a novel therapeutic target for DFU.

**Figure 5:**
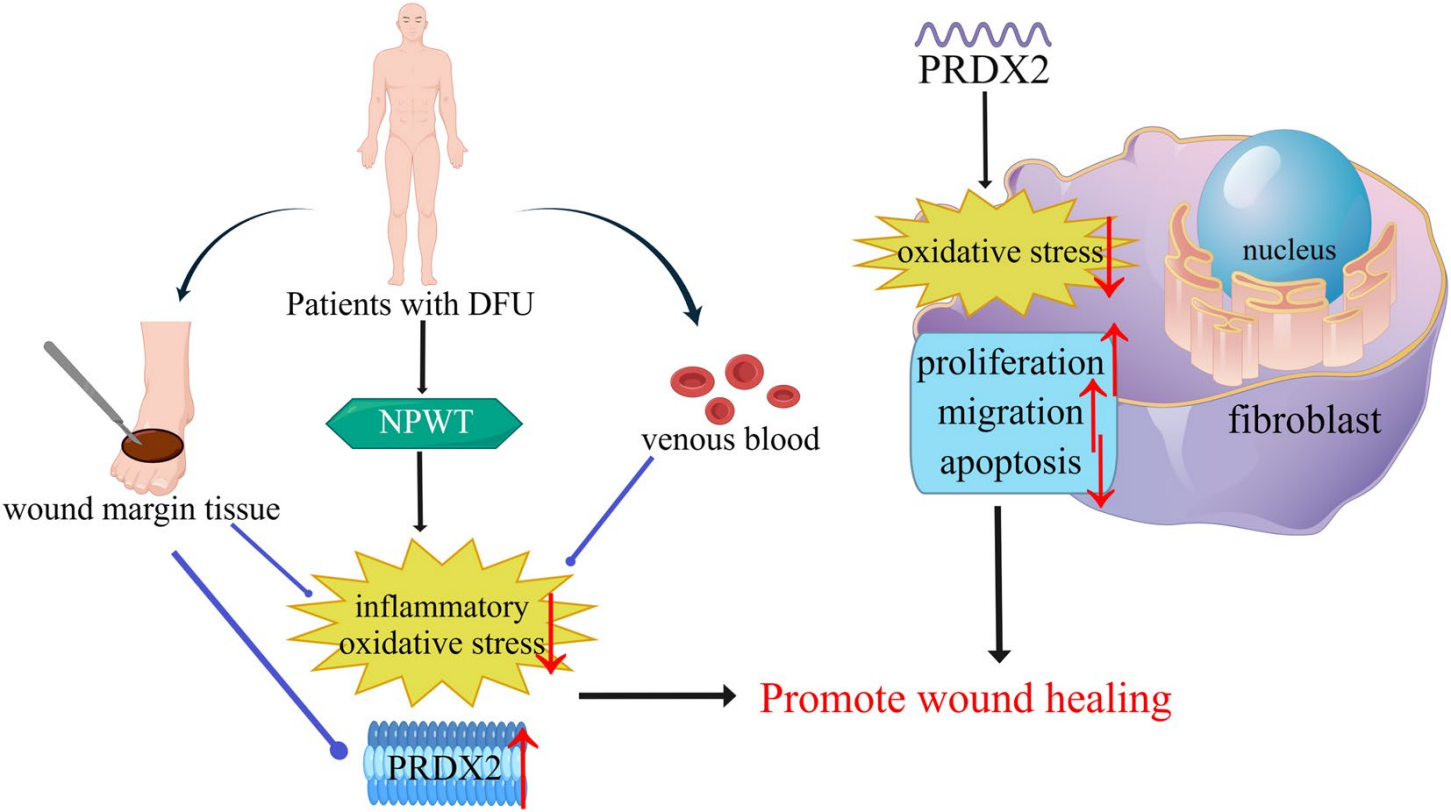
Mechanism diagram. *DFU* diabetic foot ulcer, *NPWT* negative pressure wound therapy, *PRDX2* peroxiredoxin-2.

In conclusion, the findings of this study indicate that PRDX2 promotes NHDF proliferation and migration through its antioxidant properties, which might contribute to the mechanism by which NPWT promotes chronic wound healing (Fig. 5). Furthermore, the results suggest that NPWT promotes DFU healing by increasing T-PRDX2 expression; however, its impact is lower compared to patients with chronic lower limb ulcers with normal glucose tolerance. Our data suggest that PRDX2 is a promising therapeutic target for DFU, and changes of PRDX2 in wound tissue might be associated with the prognosis of DFU patients receiving NPWT.

## Data Availability

All data generated or analysed during this study and supporting our findings are included and can be found in the manuscript. The raw data can be provided by corresponding author on reasonable request.

## References

[1] Lazzarini, P. A. *et al.* Global trends in the incidence of hospital admissions for diabetes-related foot disease and amputations: A review of national rates in the 21st century. *Diabetologia* **66**(2), 267–287. 10.1007/s00125-022-05845-9 (2023). 36512083 10.1007/s00125-022-05845-9

[2] Zhang, P. *et al.* Global epidemiology of diabetic foot ulceration: A systematic review and meta-analysis. *Ann. Med.* **49**(2), 106–116. 10.1080/07853890.2016.1231932 (2017). 27585063 10.1080/07853890.2016.1231932

[3] Zhao, X. *et al.* Decreased expression of miR-204-3p in peripheral blood and wound margin tissue associated with the onset and poor wound healing of diabetic foot ulcers. *Int. Wound J.* **20**(2), 413–429. 10.1111/iwj.13890 (2023). 35879811 10.1111/iwj.13890 PMC9885452

[4] Zhang, Y. *et al.* Global disability burdens of diabetes-related lower-extremity complications in 1990 and 2016. *Diabetes Care* **43**(5), 964–974. 10.2337/dc19-1614 (2020). 32139380 10.2337/dc19-1614

[5] Patel, S., Srivastava, S., Singh, M. R. & Singh, D. Mechanistic insight into diabetic wounds: Pathogenesis, molecular targets and treatment strategies to pace wound healing. *Biomed. Pharmacother.* **112**, 108615. 10.1016/j.biopha.2019.108615 (2019). 30784919 10.1016/j.biopha.2019.108615

[6] Rosenberger, D. C., Blechschmidt, V., Timmerman, H., Wolff, A. & Treede, R. D. Challenges of neuropathic pain: Focus on diabetic neuropathy. *J. Neural Transm. (Vienna)* **127**(4), 589–624. 10.1007/s00702-020-02145-7 (2020). 32036431 10.1007/s00702-020-02145-7 PMC7148276

[7] Huang, H. Pericyte-endothelial interactions in the retinal microvasculature. *Int. J. Mol. Sci.* **21**(19), 7413. 10.3390/ijms21197413 (2020). 33049983 10.3390/ijms21197413 PMC7582747

[8] van Netten, J. J. *et al.* Definitions and criteria for diabetic foot disease. *Diabetes Metab. Res. Rev.* **36**(Suppl 1), e3268. 10.1002/dmrr.3268 (2020). 31943705 10.1002/dmrr.3268

[9] Peskin, A. V., Pace, P. E. & Winterbourn, C. C. Enhanced hyperoxidation of peroxiredoxin 2 and peroxiredoxin 3 in the presence of bicarbonate/CO_2_. *Free Radic. Biol. Med.* **145**, 1–7. 10.1016/j.freeradbiomed.2019.09.010 (2019). 31521665 10.1016/j.freeradbiomed.2019.09.010

[10] Yu, Y. *et al.* Dihydroartemisinin enhances the anti-tumor activity of oxaliplatin in colorectal cancer cells by altering PRDX2-reactive oxygen species-mediated multiple signaling pathways. *Phytomedicine* **98**, 153932. 10.1016/j.phymed.2022.153932 (2022). 35104762 10.1016/j.phymed.2022.153932

[11] Kang, S. W., Rhee, S. G., Chang, T. S., Jeong, W. & Choi, M. H. 2-Cys peroxiredoxin function in intracellular signal transduction: Therapeutic implications. *Trends Mol. Med.* **11**(12), 571–578. 10.1016/j.molmed.2005.10.006 (2005). 16290020 10.1016/j.molmed.2005.10.006 PMC7185838

[12] Li, J. *et al.* PRDX2 protects against atherosclerosis by regulating the phenotype and function of the vascular smooth muscle cell. *Front. Cardiovasc. Med.* **8**, 624796. 10.3389/fcvm.2021.624796 (2021). 33791345 10.3389/fcvm.2021.624796 PMC8006347

[13] Chen, Y. *et al.* Isorhapontigenin attenuates cardiac microvascular injury in diabetes via the inhibition of mitochondria-associated ferroptosis through PRDX2-MFN2-ACSL4 pathways. *Diabetes* **72**(3), 389–404. 10.2337/db22-0553 (2023). 36367849 10.2337/db22-0553

[14] Lee, S. C., Na, Y. P. & Lee, J. B. Expression of peroxiredoxin II in vascular tumors of the skin: A novel vascular marker of endothelial cells. *J. Am. Acad. Dermatol.* **49**(3), 487–491. 10.1067/s0190-9622(03)01485-3 (2003). 12963914 10.1067/s0190-9622(03)01485-3

[15] Han, Y. H. *et al.* Deletion of peroxiredoxin II inhibits the growth of mouse primary mesenchymal stem cells through induction of the G0/G1 cell-cycle arrest and activation of AKT/GSK3β/β-catenin signaling. *In Vivo* **34**(1), 133–141. 10.21873/invivo.11754 (2020). 31882472 10.21873/invivo.11754 PMC6984073

[16] Martin, P. & Nunan, R. Cellular and molecular mechanisms of repair in acute and chronic wound healing. *Br. J. Dermatol.* **173**(2), 370–378. 10.1111/bjd.13954 (2015). 26175283 10.1111/bjd.13954 PMC4671308

[17] Pastar, I. *et al.* Epigenetic regulation of cellular functions in wound healing. *Exp. Dermatol.* **30**(8), 1073–1089. 10.1111/exd.14325 (2021). 33690920 10.1111/exd.14325 PMC8324509

[18] Liu, Y. *et al.* Fibroblasts: Immunomodulatory factors in refractory diabetic wound healing. *Front. Immunol.* **13**, 918223. 10.3389/fimmu.2022.918223 (2022). 35990622 10.3389/fimmu.2022.918223 PMC9391070

[19] Kruse, C. R., Singh, M., Sørensen, J. A., Eriksson, E. & Nuutila, K. The effect of local hyperglycemia on skin cells in vitro and on wound healing in euglycemic rats. *J. Surg. Res.* **206**(2), 418–426. 10.1016/j.jss.2016.08.060 (2016). 27884338 10.1016/j.jss.2016.08.060

[20] Peng, Y. *et al.* Ski promotes proliferation and inhibits apoptosis in fibroblasts under high-glucose conditions via the FoxO1 pathway. *Cell Prolif.* **54**(2), e12971. 10.1111/cpr.12971 (2021). 33349993 10.1111/cpr.12971 PMC7849170

[21] Zhao, X. *et al.* Changes in miroRNA-103 expression in wound margin tissue are related to wound healing of diabetes foot ulcers. *Int. Wound J.* **20**(2), 467–483. 10.1111/iwj.13895 (2023). 35837786 10.1111/iwj.13895 PMC9885465

[22] Chen, L. *et al.* A systematic review and meta-analysis of efficacy and safety of negative pressure wound therapy in the treatment of diabetic foot ulcer. *Ann. Palliat. Med.* **10**(10), 10830–10839. 10.21037/apm-21-2476 (2021). 34763444 10.21037/apm-21-2476

[23] Jia, Z. *et al.* Proteomics changes after negative pressure wound therapy in diabetic foot ulcers. *Mol. Med. Rep.* **24**(6), 834. 10.3892/mmr.2021.12474 (2021). 34608502 10.3892/mmr.2021.12474 PMC8503750

[24] Huang, C., Leavitt, T., Bayer, L. R. & Orgill, D. P. Effect of negative pressure wound therapy on wound healing. *Curr. Probl. Surg.* **51**(7), 301–331. 10.1067/j.cpsurg.2014.04.001 (2014). 24935079 10.1067/j.cpsurg.2014.04.001

[25] Chinese Diabetes Society, Chinese Society of Infectious Diseases, Chinese Society for Tissue Repair and Regeneration. Chinese guideline on prevention and management of diabetic foot. *Chin. J. Diabetes Mellitus* **11**(2), 92–108 (2019).

[26] Mu, S. *et al.* Effect of negative-pressure wound therapy on the circulating number of peripheral endothelial progenitor cells in diabetic patients with mild to moderate degrees of ischaemic foot ulcer. *Vascular* **27**(4), 381–389 (2019). 30841790 10.1177/1708538119836360

[27] Levey, A. S. *et al.* A more accurate method to estimate glomerular filtration rate from serum creatinine: a new prediction equation. Modification of diet in renal disease study group. *Ann. Intern. Med.* **130**(6), 461–470. 10.7326/0003-4819-130-6-199903160-00002 (1999). 10075613 10.7326/0003-4819-130-6-199903160-00002

[28] Yazdanpanah, L. *et al.* Prevalence and related risk factors of diabetic foot ulcer in Ahvaz, south west of Iran. *Diabetes Metab. Syndr.* **12**(4), 519–524. 10.1016/j.dsx.2018.03.018 (2018). 29602761 10.1016/j.dsx.2018.03.018

[29] Chen, L., Sun, S., Gao, Y. & Ran, X. Global mortality of diabetic foot ulcer: A systematic review and meta-analysis of observational studies. *Diabetes Obes. Metab.* **25**(1), 36–45. 10.1111/dom.14840 (2023). 36054820 10.1111/dom.14840

[30] Atkin, L. Chronic wounds: the challenges of appropriate management. *Br. J. Community Nurs.* **24**(9), S26–S32. 10.12968/bjcn.2019.24.Sup9.S26 (2019). 31479336 10.12968/bjcn.2019.24.Sup9.S26

[31] Armstrong, D. G. & Gurtner, G. C. A histologically hostile environment made more hospitable?. *Nat. Rev. Endocrinol.* **14**(9), 511–512. 10.1038/s41574-018-0073-6 (2018). 30054565 10.1038/s41574-018-0073-6

[32] Vujčić, S. *et al.* Oxidative stress and inflammatory biomarkers in patients with diabetic foot. *Medicina (Kaunas)* **58**(12), 1866. 10.3390/medicina58121866 (2022). 36557068 10.3390/medicina58121866 PMC9785583

[33] Burgess, J. L., Wyant, W. A., Abdo Abujamra, B., Kirsner, R. S. & Jozic, I. Diabetic wound-healing science. *Medicina (Kaunas)* **57**(10), 1072. 10.3390/medicina57101072 (2021). 34684109 10.3390/medicina57101072 PMC8539411

[34] Akhter, A. S. *et al.* Negative pressure wound therapy in spinal fusion patients. *Int. Wound J.* **18**(2), 158–163. 10.1111/iwj.13507 (2021). 33236841 10.1111/iwj.13507 PMC8243993

[35] Wang, G. *et al.* Negative-pressure wound therapy in a *Pseudomonas aeruginosa* infection model. *Biomed. Res. Int.* **2018**, 9496183. 10.1155/2018/9496183 (2018). 29862301 10.1155/2018/9496183 PMC5976956

[36] Zheng, S. *et al.* Negative-pressure wound therapy (NPWT) for the treatment of pacemaker pocket infection in patients unable or unwilling to undergo CIED extraction. *J. Interv. Card. Electrophysiol.* **61**(2), 245–251. 10.1007/s10840-020-00805-y (2021). 32572720 10.1007/s10840-020-00805-y

[37] Gao, J. *et al.* Negative pressure wound therapy for surgical site infections: A systematic review and meta-analysis. *J. Adv. Nurs.* **77**(10), 3980–3990. 10.1111/jan.14876 (2021). 33905552 10.1111/jan.14876

[38] Liu, Z. *et al.* Negative pressure wound therapy for treating foot wounds in people with diabetes mellitus. *Cochrane Database Syst. Rev.* **10**(10), CD010318. 10.1002/14651858 (2018). 30328611 10.1002/14651858.CD010318.pub3 PMC6517143

[39] Bellot, G. L. *et al.* MnSOD is implicated in accelerated wound healing upon Negative Pressure Wound Therapy (NPWT): A case in point for MnSOD mimetics as adjuvants for wound management. *Redox Biol.* **20**, 307–320. 10.1016/j.redox.2018.10.014 (2019). 30390545 10.1016/j.redox.2018.10.014 PMC6218638

[40] Qiu, X. *et al.* Roles of oxidative stress and Raftlin in wound healing under negative-pressure wound therapy. *Clin. Cosmet. Investig. Dermatol.* **14**, 1745–1753. 10.2147/CCID.S334248 (2021). 34848985 10.2147/CCID.S334248 PMC8612843

[41] Park, J. G. *et al.* Peroxiredoxin 2 deficiency exacerbates atherosclerosis in apolipoprotein E-deficient mice. *Circ. Res.* **109**(7), 739–749. 10.1161/CIRCRESAHA.111.245530 (2011). 21835911 10.1161/CIRCRESAHA.111.245530 PMC5474748

[42] Kim, M. H. *et al.* Peroxiredoxin 2 deficiency reduces white adipogenesis due to the excessive ROS generation. *Cell Biol. Int.* **44**(10), 2086–2093. 10.1002/cbin.11417 (2020). 32639620 10.1002/cbin.11417

[43] Chen, X. *et al.* Celastrol induces ROS-mediated apoptosis via directly targeting peroxiredoxin-2 in gastric cancer cells. *Theranostics* **10**(22), 10290–10308. 10.7150/thno.46728 (2020). 32929349 10.7150/thno.46728 PMC7481428

[44] Brinkmann, C. *et al.* Influence of glycemic status and physical fitness on oxidative stress and the peroxiredoxin system in the erythrocytes of non-insulin-dependent type 2 diabetic men. *Exp. Clin. Endocrinol. Diabetes.* **119**(9), 559–564. 10.1055/s-0031-1279712 (2011). 21667443 10.1055/s-0031-1279712

[45] Jin, X. *et al.* PRDX2 in myocyte hypertrophy and survival is mediated by TLR4 in Acute infarcted myocardium. *Sci. Rep.* **7**(1), 6970. 10.1038/s41598-017-06718-7 (2017). 28765537 10.1038/s41598-017-06718-7 PMC5539327

[46] Luo, P. *et al.* Celastrol induces ferroptosis in activated HSCs to ameliorate hepatic fibrosis *via* targeting peroxiredoxins and HO-1. *Acta Pharm. Sin. B.* **12**(5), 2300–2314. 10.1016/j.apsb.2021.12.007 (2022). 35646542 10.1016/j.apsb.2021.12.007 PMC9136576

[47] Xu, G. L., Ye, X. L., Vashisth, M. K. & Zhao, W. Z. Correlation between PRDX2 and spermatogenesis under oxidative stress. *Biochem. Biophys. Res. Commun.* **656**, 139–145. 10.1016/j.bbrc.2023.03.050 (2023). 36963350 10.1016/j.bbrc.2023.03.050

[48] Feng, A. L. *et al.* PRDX2 plays an oncogenic role in esophageal squamous cell carcinoma via Wnt/β-catenin and AKT pathways. *Clin. Transl. Oncol.* **22**(10), 1838–1848. 10.1007/s12094-020-02323-9 (2020). 32130676 10.1007/s12094-020-02323-9

[49] Zhang, S., He, J., Tang, M. & Sun, H. Prdx2 upregulation promotes the growth and survival of gastric cancer cells. *Pathol. Oncol. Res.* **26**(3), 1869–1877. 10.1007/s12253-019-00783-1 (2020). 31807984 10.1007/s12253-019-00783-1

[50] Zheng, X. *et al.* PRDX2 removal inhibits the cell cycle and autophagy in colorectal cancer cells. *Aging (Albany NY)* **12**(16), 16390–16409. 10.18632/aging.103690 (2020). 32692719 10.18632/aging.103690 PMC7485722

