# Correction: Negative pressure wound therapy promotes wound healing of diabetic foot ulcers by up-regulating PRDX2 in wound margin tissue

**DOI:** 10.1038/s41598-026-64932-8

**Published:** 2026-08-12

**Authors:** Ying Tang, Lei Liu, Ruyan Jie, Yizhong Tang, Xiaotong Zhao, Murong Xu, Mingwei Chen

**Affiliations:** 1 Department of Endocrinology, The First Affiliated Hospital of Anhui Medical University, No.218 Jixi Road, Shushan District, Hefei City, Anhui Province People’s Republic of China; 2 Department of Burns, The First Affiliated Hospital of Anhui Medical University, Hefei City, Anhui Province People’s Republic of China

Correction to: *Scientific Reports* 10.1038/s41598-023-42634-9, published online 27 September 2023

The original version of this Article contains errors in panel C of Figure 3.

As a result of an oversight during figure assembly, the images for **HG + OE-PRDX2** and **NG + si-PRDX2** group are partially overlapped.

The correct Figure 3 and accompanying legend appear below.

**Fig. 3:**
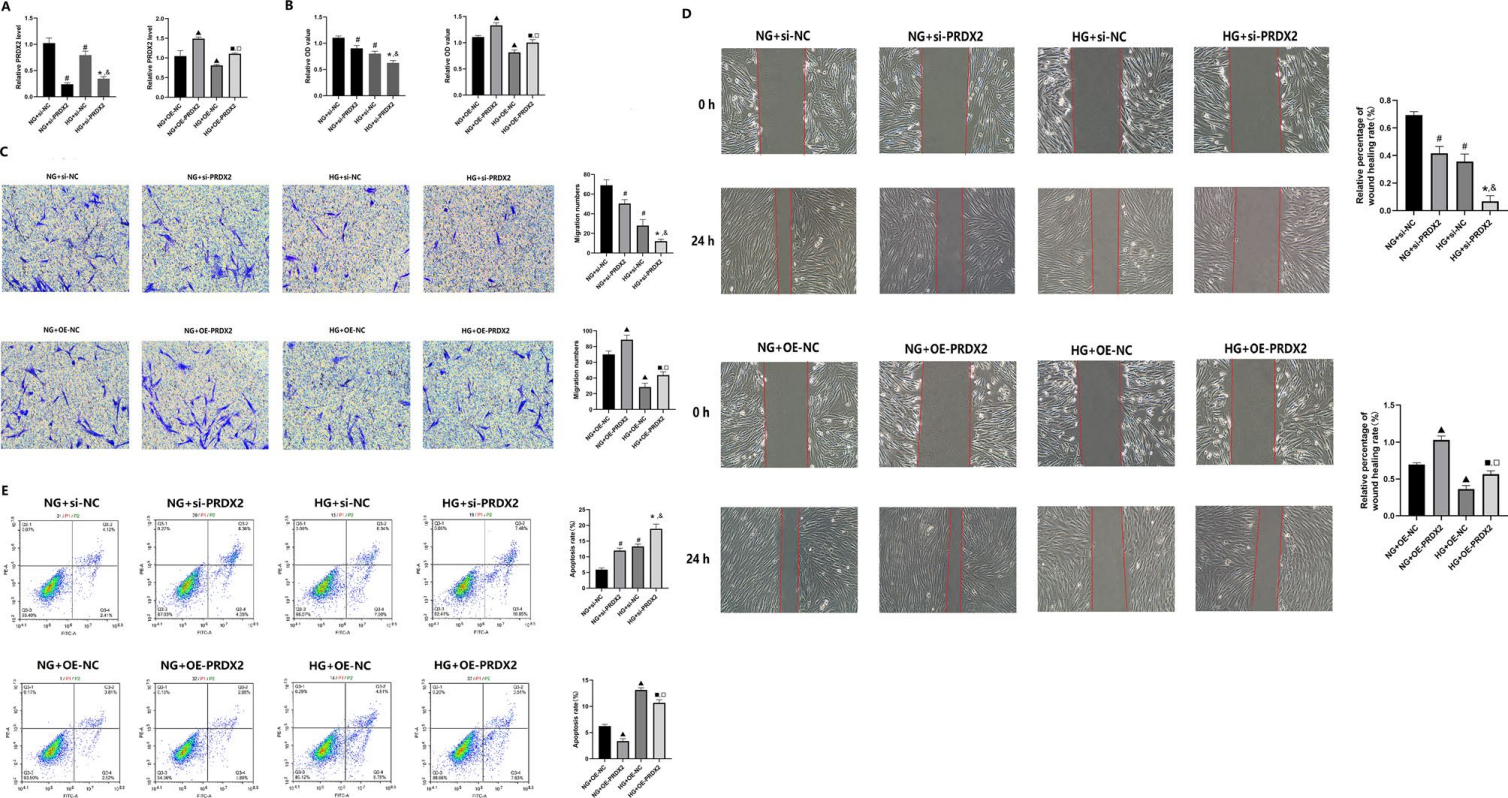
Effect of PRDX2 on proliferation, migration, and apoptosis of NHDF cells. (**A**) The mRNA expression levels of PRDX2 by qRT-PCR in NHDF cell under different conditions. (**B**) Cell proliferation was monitored by CCK8 assay. (**C** and **D**) Cell migratory capability was measured by Transwell assay and wound healing assay. (**E**) Cell apoptosis was measured by flow cytometry (Annexin V-PI); versus NG + Si-NC, #*P* < 0.05; versus HG + Si-NC, *P < 0.05; versus NG + si-PRDX2, &*P* < 0.05; versus NG + OE-NC, ▲*P* < 0.05; versus HG + OE-NC, ■*P* < 0.05; versus NG + OE-PRDX2, □*P* < 0.05.

